# Significant accumulation of nitrate in Chinese semi-humid croplands

**DOI:** 10.1038/srep25088

**Published:** 2016-04-26

**Authors:** Junyu Zhou, Baojing Gu, William H. Schlesinger, Xiaotang Ju

**Affiliations:** 1 College of Resources and Environmental Sciences, China Agricultural University, Beijing 100193, China; 2 Department of land Management, Zhejiang University, Hangzhou 310058, PR China; 3 Policy Simulation Laboratory, Zhejiang University, Hangzhou 310058, China; 4 Cary Institute of Ecosystem Studies, Millbrook, NY 12545, USA

## Abstract

Soil nitrate is important for crop growth, but it can also leach to groundwater causing nitrate contamination, a threat to human health. Here, we report a significant accumulation of soil nitrate in Chinese semi-humid croplands based upon more than 7000 samples from 141 sites collected from 1994 to 2015. In the 0–4 meters depth of soil, total nitrate accumulation reaches 453 ± 39, 749 ± 75, 1191 ± 89, 1269 ± 114, 2155 ± 330 kg N ha^−1^ on average in wheat, maize, open-field vegetables (OFV), solar plastic-roofed greenhouse vegetables (GHV) and orchard fields, respectively. Surprisingly, there is also a comparable amount of nitrate accumulated in the vadose-zone deeper than 4 meters. Over-use of N fertilizer (and/or manure) and a declining groundwater table are the major causes for this huge nitrate reservoir in the vadose-zone of semi-humid croplands, where the nitrate cannot be denitrified due to the presence of oxygen and lack of carbon sources. Future climatic change with more extreme rainfall events would increase the risk of accumulated nitrate moving downwards and threatening groundwater nitrate contamination.

The invention of Haber-Bosch N fixation (HBNF) has boosted global food production and helped to feed about half of the global population during the last century^1^. Applications of synthetic nitrogen (N) fertilizer (derived from the HBNF) or manure can increase soil N supply and substantially increase crop yields^2^. In some areas such as Africa people use far too little N for adequate food production on existing lands, while other areas, such as China, use far too much – both extremes damage the environment and threaten human well-being^3^. Even within China, the N application rates vary substantially across regions because of uneven agricultural intensification.

Over use of N fertilizer or manure results in a N surplus (N inputs minus N removed by crops) that can remain in the soil or move to other areas of the environment^4^, causing a series of negative effects, e.g., soil acidification^5^, and air and water pollution^6^. Residual N is immobilized by soil microbes or soil organic matter or fixed by clay minerals, but also exists as ammonium or nitrate in the soil matrix^7^. Because of the negative charge of nitrate, similar to soil clay in most cases, the residual nitrate is very dynamic and mobile, and can contaminate groundwater or surface water^8^. The magnitude of residual nitrate in the soil can also serve as an indicator of proper N management and for assessing the leaching risk in croplands^9^. Because nitrate present in the 0–100 cm depth interval can be recycled by roots, nitrate accumulated in this soil depth is considered to have very different environmental effects compared to that leached to >100 cm. Hereafter, we define the 0–100 cm soil profile as the root zone, and >100 cm as the vadose-zone^10^,^11^.

We are conscious of more and more reports of large accumulations of residual nitrate in different depths of soil in Chinese uplands^10^,^12^,^13^, sometimes remaining in the vadose-zone^11^ and sometimes leaching to groundwater causing nitrate contamination during the last three decades^8^,^14^. Nitrate has also accumulated in the deep vadose-zone of the Mojave Desert (California) throughout the Holocene^15^, but to our knowledge, large amounts of nitrate have never been reported to accumulate in croplands within such a short term^14^. We are interested in determining the overall magnitude and the mechanism of nitrate accumulation in the soil profile, the impacts of declining groundwater levels, and threats to groundwater contamination under future climate change.

## Results

### Overall magnitude for the nitrate accumulation in 0–4 meters

**Figure 1:**
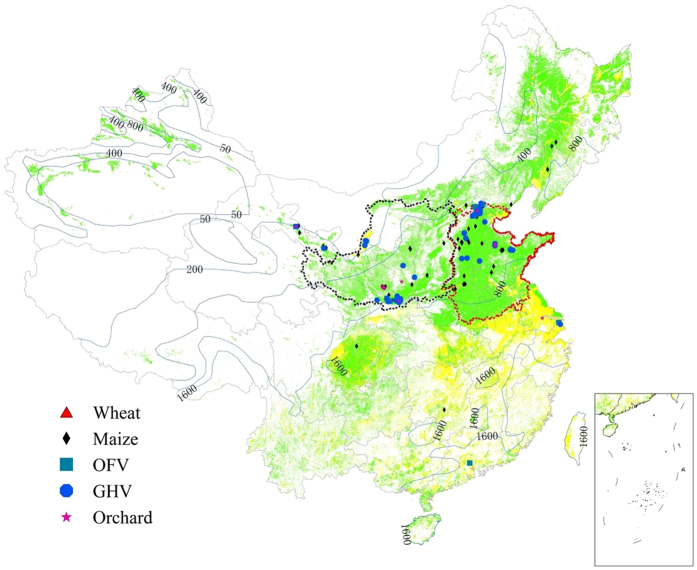
Distribution of sites for data sources of nitrate accumulation. The soil samples mainly located in the North China Plain and Loess Plateau outlined with red and black dotted lines, respectively. Dots of different colors represent the distribution of sites of nitrate accumulation for different cropping systems. The blue solid lines represent the distribution of annual average precipitation in China. Green and yellow areas in the picture represent the distribution of upland and paddy field in China. This map is generated by ArcGIS 10.3 (https://www.arcgis.com/).

**Figure 2:**
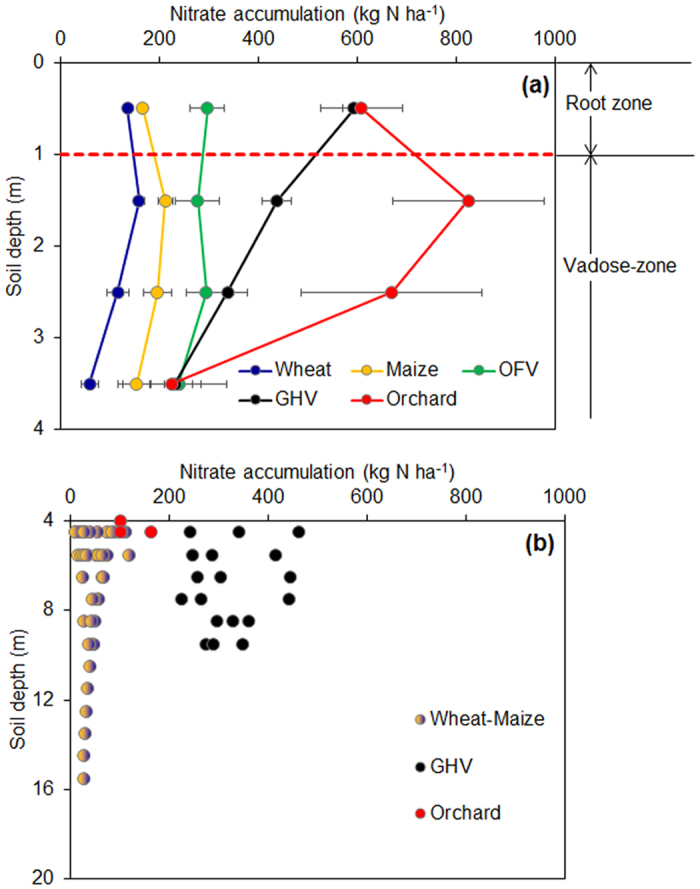
Nitrate accumulation in semi-humid croplands with different crops and soil depths. (**a**) 0–4 meter; (**b**) >4 meter. OFV, open field vegetable; GHV, greenhouse vegetable. Data points represent the nitrate accumulation in each meter interval of soil depth, and error bars represent the standard errors of the nitrate accumulation.

We collected more than 7000 analyses from 141 sites reported in literature from 1994 to 2015 for the nitrate accumulation in 0–4 meters depth of the soil profile across Chinese uplands (also including many from our own sampling and published measurements) (Fig. 1). We cataloged the data into five cropping systems, i.e. wheat, maize, open-field vegetables (OFV), solar plastic-roofed greenhouse vegetables (GHV) and orchard, mainly based on their different N inputs (Table S1) and management practices. Despite the fact that these nitrate data were obtained in different years and sites with different fertilization and management regimes, the analysis of the data shows the general magnitude of soil nitrate accumulation in Chinese uplands, and enhances our knowledge of the behavior of nitrate in semi-arid and semi-humid ecosystems, where the evapotranspiration is higher than precipitation^15^. We found extremely large amounts of nitrate accumulated in the 0–4 meters profile of Chinese upland soils (Fig. 2a, Table S2 and Fig. S1), which were 453 ± 39, 749 ± 75, 1191 ± 89, 1269 ± 114, 2155 ± 330 kg N ha^−1^ in wheat, maize, OFV, GHV and orchard, respectively, with significant variations by crop systems and depths. About 70% of the nitrate is distributed in the soil layers deeper than the 1-meter root zone, which is usually out of the zone of intensive biological activity^15^. This nitrate has a high risk of moving out of the vadose-zone to groundwater. Similar observations were also reported for the nitrate concentration in arid and semi-arid desert sites in the western United States, where nitrate followed the conservative solute accumulation profiles of chlorine (Cl^−^) rather than the expected progressive nutrient depletion with depth^15^.

### Overall magnitude for the nitrate accumulation in soils deeper than 4 meters

Data for nitrate in upland soils deeper than 4 meters are rare due to the difficulty of sampling. Fortunately, we also collected nitrate data in several studies (including our own published work) that measured depths deeper than 4 meters, but usually less than 20 meters (Fig. S2). We plotted all the available data in Fig. 2b to give a general picture of nitrate accumulation >4 meters depth. We combined nitrate data from wheat and maize fields because the samples in these studies were taken from winter wheat-summer maize rotation systems (double cropping systems in one year). Despite large variation at all depths, the nitrate accumulation in soil >4 meters is comparable to that from the 0–4 meters soil. GHV and orchard have much higher nitrate accumulation in the soil profile deeper than 4 meters compared to wheat and maize rotation.

A 15-year long-term field experiment (established in 1998, and sampled in 2012; see ref.^11^ for a detailed description of methods) of the winter wheat-summer maize double cropping rotation system in the North China Plain reported 119, 541 and 4138 kg N ha^−1^ nitrate accumulation in 0–12 m soil profile with annual N applications of 0, 200, 600 kg N ha^−1^, respectively^11^ (Fig. S2). This study illustrated large nitrate accumulation with excessive N application. The denitrification potential is very low in deep soil (>1 meter) in the vadose-zone, mainly owing to the lack of carbon sources and an oxic environment^11^. Therefore, the accumulated nitrate would gradually move downward to the deeper vadose-zone, and finally to the shallow groundwater, carried during years of exceptional rainfall and soil water percolation^11^.

### Influencing factors on nitrate accumulation

**Figure 3:**
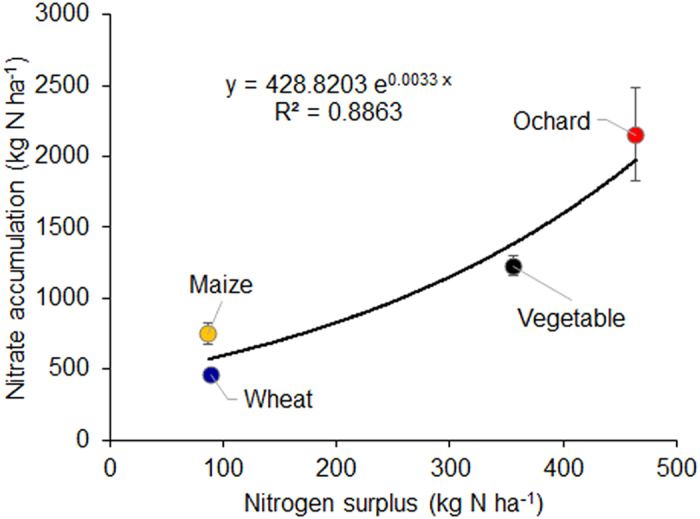
The relationship between N surplus and accumulated nitrate in 0–4 m soil profile of different cropping systems. Nitrate accumulation on the y axis represents the cumulative nitrate accumulation in the 0–4 meter soil profile. Error bars represent the standard errors of the nitrate accumulation. The exponential line represents the non-linear relationship between N surplus and nitrate accumulation. Vegetable includes the data from both open-field and greenhouse cultivation. Nitrate data are from the synthesis of this study, and the N surplus data are from ref. (23).

**Figure 4:**
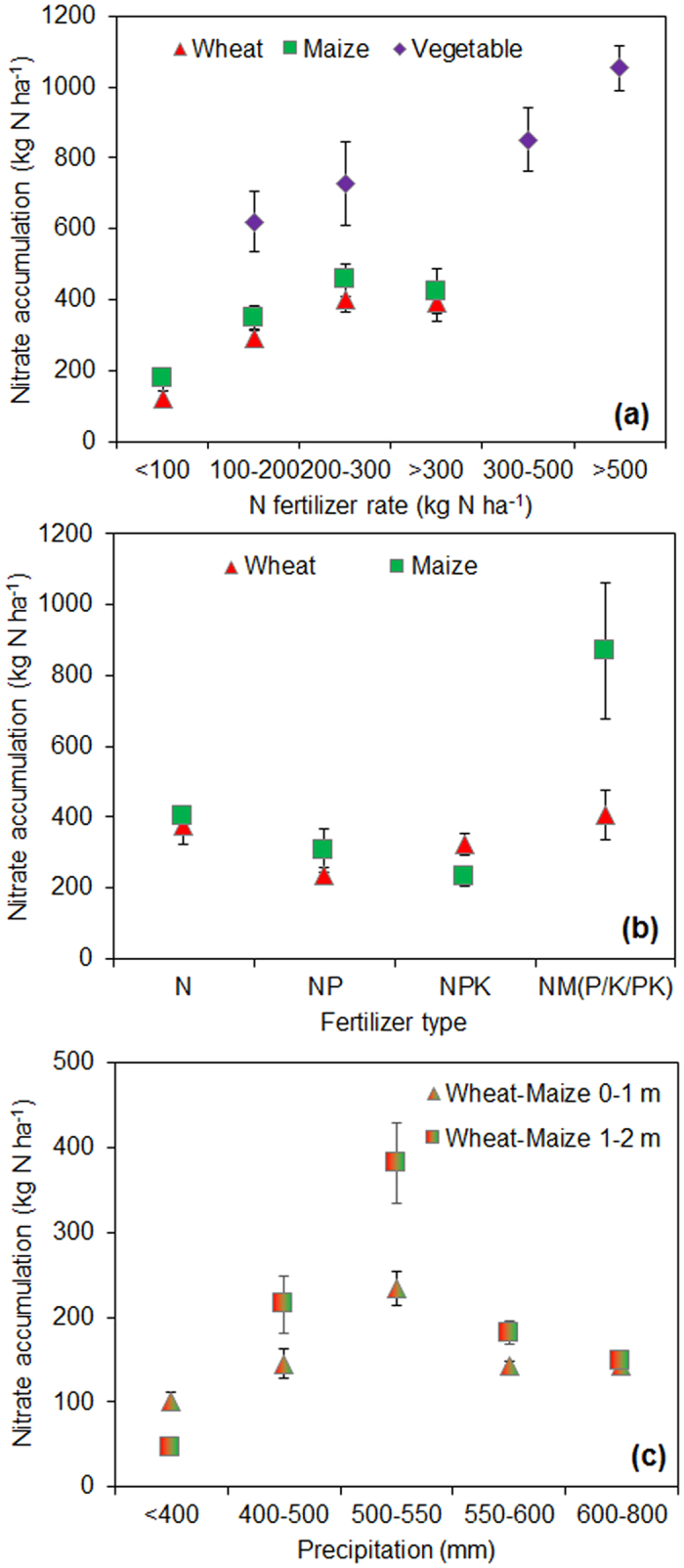
Factors influencing the nitrate accumulation in semi-humid croplands. (**a**) N fertilizer rate, the nitrate accumulation refers to 0–2 meter soil. (**b**) Fertilizer types, the nitrate accumulation in 0–2 meter soil. N represents only N fertilizer applied; NP represents both N and P fertilizers applied; NPK represents N, P and K fertilizers applied; NM (P/K/PK) represents both N fertilizer and manure applied, together with P, K or P and K fertilizers. (**c**) Precipitation.

In the above analysis, we found crop systems and land use types have significant effects on the soil nitrate accumulation at different depths. To further identify the influencing factors on nitrate accumulation, we test the effects of N surplus, N fertilizer rate, the use of other nutrients, and precipitation on the nitrate accumulation. The amount of accumulated nitrate in 0–4 meters was significantly correlated with the surplus of N in different cropping systems, and high N surplus enhances the nitrate accumulation (Fig. 3). Similarly, soil nitrate increases significantly in 0–2 meter deep layer with the rate of fertilizer N application (Fig. 4a) (here we can only plot 0–2 m data in wheat, maize and OFV + GHV because of limited data in other layers and orchard). When the fertilizer N rate is lower than 100 kg N ha^−1^, the nitrate accumulation is significantly lower than that at higher N rates, and these results are robust under different cropping systems. Currently, the recommended N applications are 150–250 kg N ha^−1^ in wheat or maize, and 150–300 kg N ha^−1^ and 150–250 kg N ha^−1^ in vegetable and orchard crops, respectively, in Chinese intensive managed cropping systems^16^. These recommended N rates have already caused high nitrate accumulation, yet even higher N application rates are common in farmers’ practices. These recommended N rates have frequently exceeded the economic optimum N rate or maximum yield N rate in Chinese croplands. The diminishing returns of increasing N input lead to a large N surplus that enhances the nitrate accumulation in soil profile^2^.

Numerous studies have shown that little nitrate would accumulate or leach if the N fertilizer application is less than that for optimum or maximum crop yield; however, the residual nitrate accumulation and leaching increase sharply once the N fertilizer rate is higher than the optimum^4^,^17^,^18^. For example, the long-term experiments at the Rothamsted station showed no significant increase of nitrate leaching with the N fertilizer lower than the economic optimum N rate (144 kg N ha^−1^), but sharply increased nitrate leaching at higher applications^18^. Raun and Gordon found that soil nitrate accumulation would increase sharply when the N rate is 23 kg N ha^−1^ higher than the N rate that is associated with maximum crop yield^17^. Therefore, strict controls of the N application in each crop season are crucial to reduce of nitrate accumulation and leaching.

Often balanced application of other nutrients including P and K with N can significantly reduce the nitrate accumulation compared to application of only N fertilizer (Fig. 4b). The crop uptake of applied N increases when combined with P and K fertilizers, hence reducing the N surplus in soil-crop systems. Here we can only plot 0–2 m data in wheat and maize because there are few data for other layers and other cropping systems. Surprisingly, we found a higher nitrate accumulation under the combined use of both manure and synthetic N fertilizer compared to the use of synthetic N fertilizer alone. We regard this as a “carry over” effect^19^ in manure fertilization regimes which result in high total N supply by inadequate accounting for the N released from prior seasons^20^. We must pay attention to precise estimation of N supply when using manure together with synthetic N fertilizer to avoid over supply of N and the potential high risk to nitrate accumulation and leaching.

We found that the highest nitrate accumulation occurred with annual precipitation of 500–550 mm (Fig. 4c), lower or higher amounts significantly reducing the soil nitrate accumulation. Here we plot 0–1 m and 1–2 m data in wheat and maize together because there are few data in other layers and for orchards, and we did not plot vegetable fields since they are normally irrigated and less affected by precipitation. In arid and semi-arid regions (<400 mm), croplands usually receives less N fertilizer owing to low productivity limited by low water supply, thus requiring less N input. In contrast, in humid regions (>800 mm), despite more N fertilizer used, the sufficient precipitation would cause nitrate runoff, leaching, or denitrification to N_2_ or N_2_O^4^. Areas with over 800 mm precipitation are considered humid regions, where the dominant land use type is paddy fields. Thus, we seldom found nitrate accumulation in Southern China where rice is cultivated although excessive N fertilizer also used there. The majority of the N surplus in paddy fields is lost to the environment through runoff and denitrification^4^. In sum, the high nitrate accumulation in the soil profile is mostly detected in semi-humid regions with precipitation between 400 and 800 mm. That is why these accumulations are reported in North China, especially in North China Plain and Loess Plateau (Fig. 1).

## Discussion

### Mechanism of nitrate accumulation in the soil profile

Previous work found that ammonium-based N fertilizers or urea (which provides about 90% of Chinese N fertilizers) applied to soil are easily volatilized in the form of ammonia and nitrified to nitrate within 0.5–2 weeks in the favorable temperature and moisture conditions of calcareous soils in the semi-arid and semi-humid regions of Northern China^21^. The ammonium N content in the soil profile remains at a low and constant level around 5 mg N kg^−1^ in the cultivated layer (0–30 cm depth) except for a short time after N fertilization. Nitrate contents in soil profile were significantly altered by the rate of applied N, with more nitrate at higher fertilizer applications^21^. Almost all the applied N fertilizer can be recovered as nitrate after nitrification in soil incubation experiments^22^. Thus, (1) when the N fertilizer application rate exceeds crop demand, there is surplus N in the soil-crop system, leading to much residual N in the soil profile after each crop^4^,^10^,^12^,^13^,^21^,^23^; (2) a large proportion of residual N exists as nitrate due to the high nitrification rates in these soils^22^,^24^; (3) The residual nitrate can accumulate in the soil profile due to the N surplus occurring in every crop growth season^10^,^11^,^21^; (4) The accumulated nitrate easily leaches to the subsoil or deep vadose-zone after intensive rainfalls in the summer season (characterized by high temperature and moisture from July to September, contributing about 70% of the annual precipitation) or excessive flood irrigation^4^,^10^,^12^,^13^,^14^,^21^,^24^.

Nitrification of ammonium-based N fertilizer or urea, which causes nitrate to move from cultivated layers (0–20 or 30 cm top soil layer) to the subsoil are the cause of strong acidification in the top cultivated layers in Chinese croplands^5^. Soil water-filled pore space (WFPS) contains enough oxygen and seldom reaches the necessary anaerobic conditions for denitrification to convert the nitrate to N_2_ or N_2_O^25^. The processes immobilizing nitrate in soil organic matter (SOM) or soil microbes are weak due to low SOM and lack of significant carbon sources^26^. Therefore, a large amount of nitrate accumulates in Chinese semi-humid croplands, which are characterized by a high N surplus, low carbon content, strong mineralization and nitrification abilities, and weak immobilization and denitrification abilities. All above processes make these kinds of soils accumulate nitrate in the profile.

### The interaction between nitrate accumulation and groundwater table decline

Groundwater pollution, eutrophication of surface water, and coastal red tides have become worse in China since the 1980s, and N is one of the likely contributors^8^,^27^. Although it is difficult to build a causal relationship between N fertilizer input, soil nitrate accumulation and water pollution on the national scale, they should be closely linked if we consider their synchronization on both spatial and temporal scales^27^. Here, we took the North China Plain (NCP) as a case study to illustrate the nitrate accumulation in soil, movement in the vadose-zone, pollution of groundwater, and concomitant shallow groundwater decline during the past three decades.

**Figure 5:**
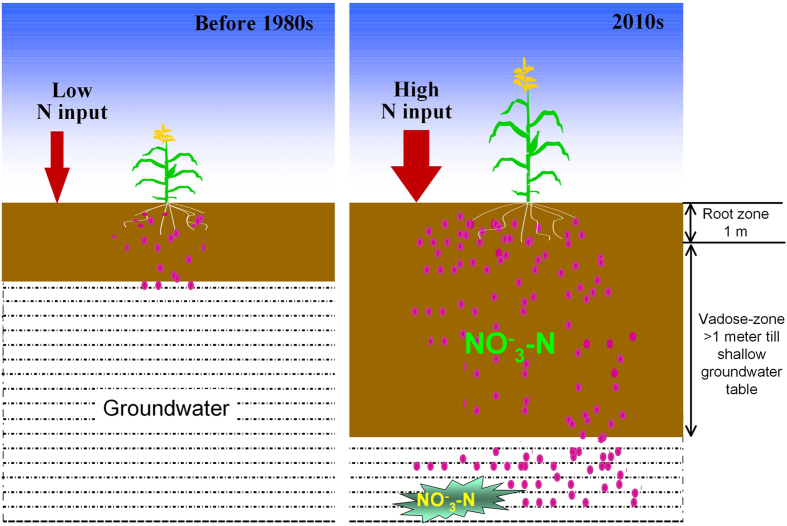
Farmework of nitrate accumulation in croplands of the North China Plain for two different periods: before 1980s and 2010s with low and high N fertilizer input, respectively.

The NCP is a typical sub-humid intensive agricultural region with excessive N input and over exploitation of groundwater for irrigation. Historically, the perennial groundwater table is around 5-m depth, although summer rainfall can raise the level of groundwater. Before the 1980s, the N fertilizer rate was low and N surplus was seldom observed, therefore, little soil nitrate accumulation or groundwater pollution were found although the groundwater table was near the root zone (Fig. 5). However, the exploitation of groundwater in last three decades due to irrigation of agricultural lands has sharply reduced the level of groundwater in the NCP. Previous studies have well documented that the groundwater table in the NCP has fallen from around 5 m in 1960 to around 20–30 m in 2010 with an annual rate of decline of 0.5–0.7 m, and that this trend is still continuing with intensification of agriculture and climatic change (slight decreasing precipitation)^28^. This decline has formed a thick vadose-zone, in which few carbon sources and the oxic conditions inhibit the denitrification. The thick vadose-zone has become a reservoir that captured a huge nitrate accumulation and changed the behavior of nitrate movement (Fig. 5). Because of the thick vadose-zone, the accumulated nitrate since 1990s could not rapidly move to the groundwater, and only moves downward during heavy rainfalls in summer season. Thus, the N surplus, soil nitrate accumulation, decline of groundwater level and groundwater nitrate contamination in China co-occur after 1990s.

### Threats to groundwater contamination under future climate change

Although the situation of soil nitrate accumulation in China is already serious, future climatic change could make it worse with more extreme rainfall events. Annual rainfall in Northern China has declined from ~600 mm in 1960s to ~500 mm in 2010s; however, extreme rainfall events increased during this period, especially in summer^28^. These trends are likely to continue in this century^28^,^29^. During each rainstorm, the newly formed nitrate and accumulated nitrate would go deeper with water to vadose-zone or shallow groundwater. These significant accumulations of nitrate in semi-humid cropland soils could become a major threat to the groundwater quality.

**Figure 6:**
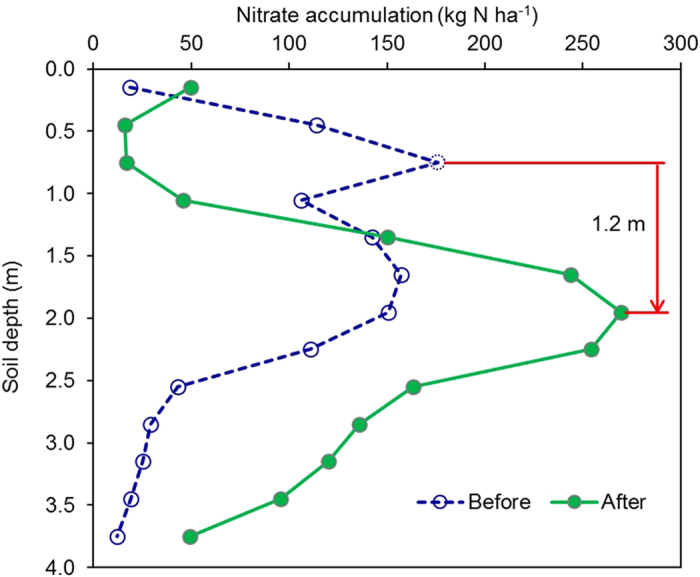
Movement of nitrate in 0–4 m soil profile in a field experiment site: before and after the heavy rainfall season of the summer in 2006 in Dongbeiwang, Haidian District, suburb of Beijing (also see ref. (10) for detail description of the field experiment).

We have conducted a 6-year-experiment (1999–2006, see ref.^10^ for the details describes of methods) with winter wheat and summer maize rotation in the NCP^10^, which can provide direct evidence to the above phenomena. The large amount of N surplus when farmers’ practices applied a total of 600 kg N ha^−1^ yr^−1^ led to soil nitrate accumulations of 201, 437, 481, 918 and 1324 kg N ha^−1^ from 1999 to 2003, respectively, in the 0–200 cm depth of soil. Summer rainfall in 2006 was significantly stronger than for the same period in 2000–2005, with two heavy rainfall events >70 mm (Fig. S3). Fortunately, we recorded the nitrate accumulation before and after the heavy rainfalls, which reveal the movement of nitrate through the vadose-zone (Fig. 6). The nitrate accumulation increased from 1068 ± 190 to 1583 ± 319 kg N ha^−1^ and the peak of nitrate accumulation moved downward 120 cm between two samples of soil at a depth of 0–4 meters before and after the heavy rainfalls. In a normal year, nitrate accumulates in the different depths of the soil profile, but in an extreme rainfall year, the accumulated nitrate would move down a large distance. Our study also showed that the leaching of nitrate depends not only on the total amount of annual precipitation, but also the intensity of rainfalls.

This study gives an overall picture of the significant accumulation of soil nitrate in China, and it casts doubt on the sustainable development of intensive agriculture. These issues are not only serious in China, but also have a great significance in other countries, especially countries that are increasing N fertilizer use. Because the nitrate could accumulate in the deep vadose-zone and groundwater for a long-term^30^, even if we stop the over use of N fertilizer immediately, these cumulative nitrate deposits would still exist for decades^30^. Chinese scientists, public and government have realized these serious problems, and a series of policies (e.g., zero increase of fertilizer, Clean Water Act, etc.) have been launched. We hope these policies can mitigate the nitrate pollution and push forward the development of sustainable agriculture in China.

## Methods

### Data collection on soil nitrate

To understand the overall pattern of nitrate accumulation in Chinese croplands, a comprehensive literature analysis was conducted through searching ISI Web of Science, Google Scholar and China National Knowledge Infrastructure (CNKI) (1980–2015). We reviewed more than 2700 published papers and chose 206 of them, retrieving over 7000 data records (extended reference list is provided in Supplementary Information), referring to the soil nitrate accumulation and related factors, such as soil-climatic factors, fertilizer rate and types. To quantify the nitrate accumulation in soil profile, the following criteria were applied: (i) the sampling interval is 20 cm or 30 cm depth (some studies in 40 or 60 cm depth in deep soil layer), and the soil bulk density is reported and usually determined by the ring-cut method in each layer 31,32; (2) soil samples are extracted by standard methods with appropriate extracting solutions (2M KCl, 1M KCl or 0.01M CaCl_2_)^31^,^32^; (3)nitrate concentration (mg N L^−1^) is measured using the standard methods such as automated continuous flow analyzer^32^ and ultraviolet spectrophotometry^33^; (4) the cumulative amount of soil nitrate (kg N ha^−1^) can be calculated by soil depth, soil bulk density and soil nitrate content(mg N kg^−1^), and can be reported by the interval of 1-m depth of soil (e.g., 0–1 m, 1–2 m etc.)^10^,^12^,^13^,^21^,^24^; (5) the cropping systems on the sampling sites are reported, e.g., wheat, maize, open field vegetables (OFV), solar plastic-roofed greenhouse vegetables (GHV) and orchard; (6) detailed geographical location of the sampling sites are reported (Fig. 1). Based on the above dataset, we can report the variations of nitrate accumulation under different soil depths and cropping systems.

### Influencing factors on soil nitrate

To understand the factors affecting nitrate accumulation in soil profile, we include the features of N fertilizer rate, fertilizer types and precipitation accompanying the data for soil nitrate accumulation. Most of the sampling locations were in long-term experimental sites or in farmers’ fields with consistent long-term practices. We believe that these treatments illustrate the influence of various factors on long term nitrate accumulation. Fertilizer treatments include N, phosphorus (P), potassium (K) and manure or the different modes of combinations, and these fertilizers are converted to the amount of nutrients of N, P_2_O_5_ and K_2_O in the fertilizer in units of kg ha^−1^. Totally, we collected 1779, 1390, and 1599 datasets for N fertilizer rate, fertilizer type and precipitation, respectively. The nitrate amount in different soil layers of different cropping systems in the dataset was regarded as the independent variable.

## Additional Information

**How to cite this article**: Zhou, J. *et al*. Significant accumulation of nitrate in Chinese semi-humid croplands. *Sci. Rep.* **6**, 25088; doi: 10.1038/srep25088 (2016).

## Supplementary Material

Supplementary Information
