# Supplementary material for: Significant accumulation of nitrate in Chinese semi-humid croplands
Source: Sci Rep. 2016 Apr 26;6:25088. doi: 10.1038/srep25088 (PMC4844977; doi:10.1038/srep25088)
Supplement: Supplementary Information [file srep25088-s1.doc]

**Supplementary Information**

**Significant accumulation of nitrate in Chinese semi-humid croplands**

Junyu Zhou^1*^, Baojing Gu^2,3*^, William H. Schlesinger^4^, Xiaotang Ju^1^

^1^College of Resources and Environmental Sciences, China Agricultural University, Beijing 100193, China;

^2^Department of land Management, Zhejiang University, Hangzhou 310058, PR China;

^3^Policy Simulation Laboratory, Zhejiang University, Hangzhou 310058, PR China;

^4^Cary Institute of Ecosystem Studies, Millbrook, NY 12545, USA**;**

^*^ These authors contributed equally to this work

**Corresponding Author: Xiaotang Ju**

College of Resources and Environmental Sciences, China Agricultural University, Beijing 100193, PR China. Tel.: +86 10 62732006; fax: +86 10 62731016. E-mail: [juxt@cau.edu.cn](mailto:juxt@cau.edu.cn)

**Table S1. Survey results of farmers’ practices regarding N fertilizer rate and N surplus in different cropping systems of China^1^**

| Cropping systems | No. of interview | Yield (Mg ha^-1^) | Fertilizer N (kg N ha^-1^) | Manure N (kg N ha^-1^) | Aboveground uptake (kg N ha^-1^) | N surplus (kg N ha^-1^) |
| --- | --- | --- | --- | --- | --- | --- |
| Wheat | n=4554 | 4.9±2.0^b^ | 197±134 | 15±55 | 123 | 89 |
| Maize | n=4522 | 7.4±2.7 | 231±142 | 18±52 | 162 | 87 |
| OFV+GHV^a^ | n=3889 | 36.0±36.1 | 383±263 | 56±145 | 83 | 356 |
| Orchard | n=6863 | 36.7±19.7 | 550±381 | 42±99 | 128 | 464 |

^a^ OFV+GHV=Open field vegetable + Greenhouse vegetable. ^b^ Mean±SD. N surplus= Synthetic N + Manure N − Aboveground uptake. The survey on farmers’ plot scale was conducted in 2009, including 2346 villages in 27 provinces of China. For each cropping system, three to five counties per province, and three towns per county, two villages per town, and 8-10 households per village were randomly selected and interviewed. **D**ata retrieved from ref (1).

**Table S2 Magnitudes of nitrate accumulation in 0-4 m soil profile of different cropping systems (kg N ha^−1^**)

| Soil depth(m) | Wheat | Maize | OFV | GHV | Orchard |
| --- | --- | --- | --- | --- | --- |
| 0-1  1-2  2-3  3-4 | 135±4(625)  158±11(214)  116±22(61)  59±17(33) | 166±7(520)  212±15(248)  196±29(91)  154±28(41) | 297±35(53)  277±45(27)  295±41(16)  239±27(15) | 594±24(251)  438±30(119)  338±40(13)  232±51(9) | 609±84(48)  825±153(24)  670±182(22)  225±110(20) |
| 0-4 | 453±39(112) | 749±75(68) | 1191±89(22) | 1269±114(20) | 2155±330(23) |

Data are Mean±SE in the table. The number of data is given in parentheses.

**Fig. S1** **Nitrate accumulation in 0-4 m soil profile of different cropping systems.** The blue and red lines in the boxes represent median and mean values; lower and upper edges, bars and dots in or outside represent the 25th and 75th, 5th and 95th, and <5th and >95th percentiles of all data, respectively.

**
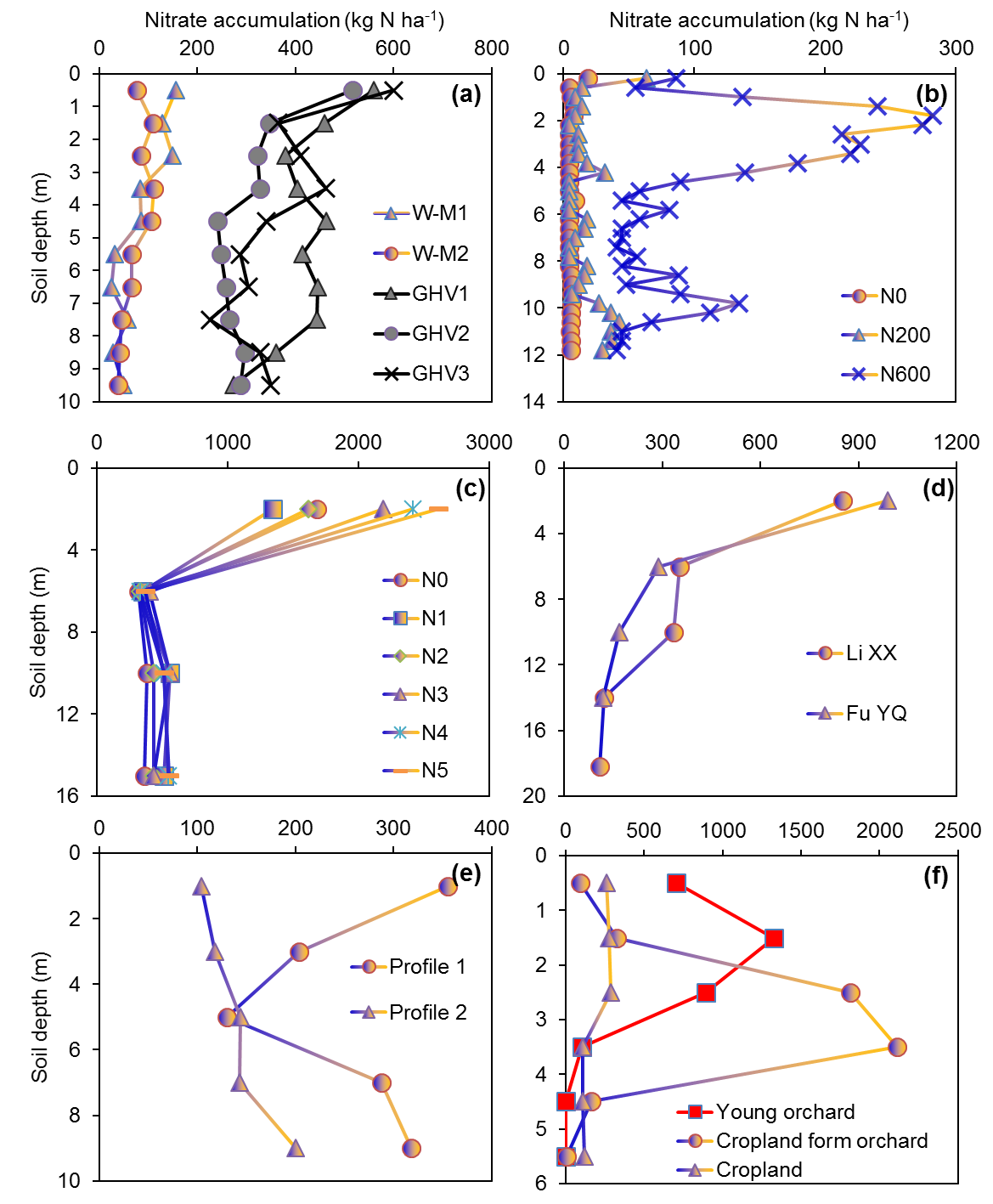
**

**Fig. S2** **Nitrate accumulation in deep vadose-zone in different cropping systems**. **a**: The sampling site is located in Dingzhou, Hebei (38.52°N, 114.99°E),in the North China Plain (NCP) with wheat and maize double cropping system. We determined the nitrate accumulated in the 0-10 m soil in two plots of wheat-maize rotation and 3 plots of GHV in farmers’ fields^2^. **b**: The long term experiment in Luancheng, Hebei (37.83°N, 114.67°E), in the NCP with wheat and maize double cropping system, the N fertilizer rate at 0, 200 and 600 kg N ha^-1^ yr^-1^ representing the control, optimum N rate and farmers’ practices since 1998, and the 0-12 m soil profile was sampled in 2012 to determine the nitrate accumulation^3^. **c**: The sampling site is located in Luancheng, Hebei (37.50°N, 114.40°E) in the NCP with wheat and maize double cropping system. The N fertilizer rate at 0, 100, 200, 400, 600 and 1500 kg N ha^-1^ before sowing the summer maize, and sampling after only one season of summer maize^4^. **d**: The sampling site is located in Luancheng, Hebei (37.90°N, 114.64°E) and (37.83°N, 114.67°E) in the NCP with perennial wheat and maize double cropping system, with the sampling depth of 16 m and 20.4 m and sampling time of 2004 in farmers’ fields^5,6^. **e**: The sampling site is located in Yangling, Shannxi (34.26°N, 108.95°E) in the Loess Plateau with wheat and maize double cropping system, with the sampling depth of 10 m in farmers’ fields^7^. **f**: The sampling site is located in Changwu, Shannxi (35.24°N, 107.69°E) in the Loess Plateau. This research determined the nitrate accumulated in 0-6 m soil profile of young orchard (15 year), cropland converted from orchard (3 year) and cropland (over 25 year), respectively^8^.


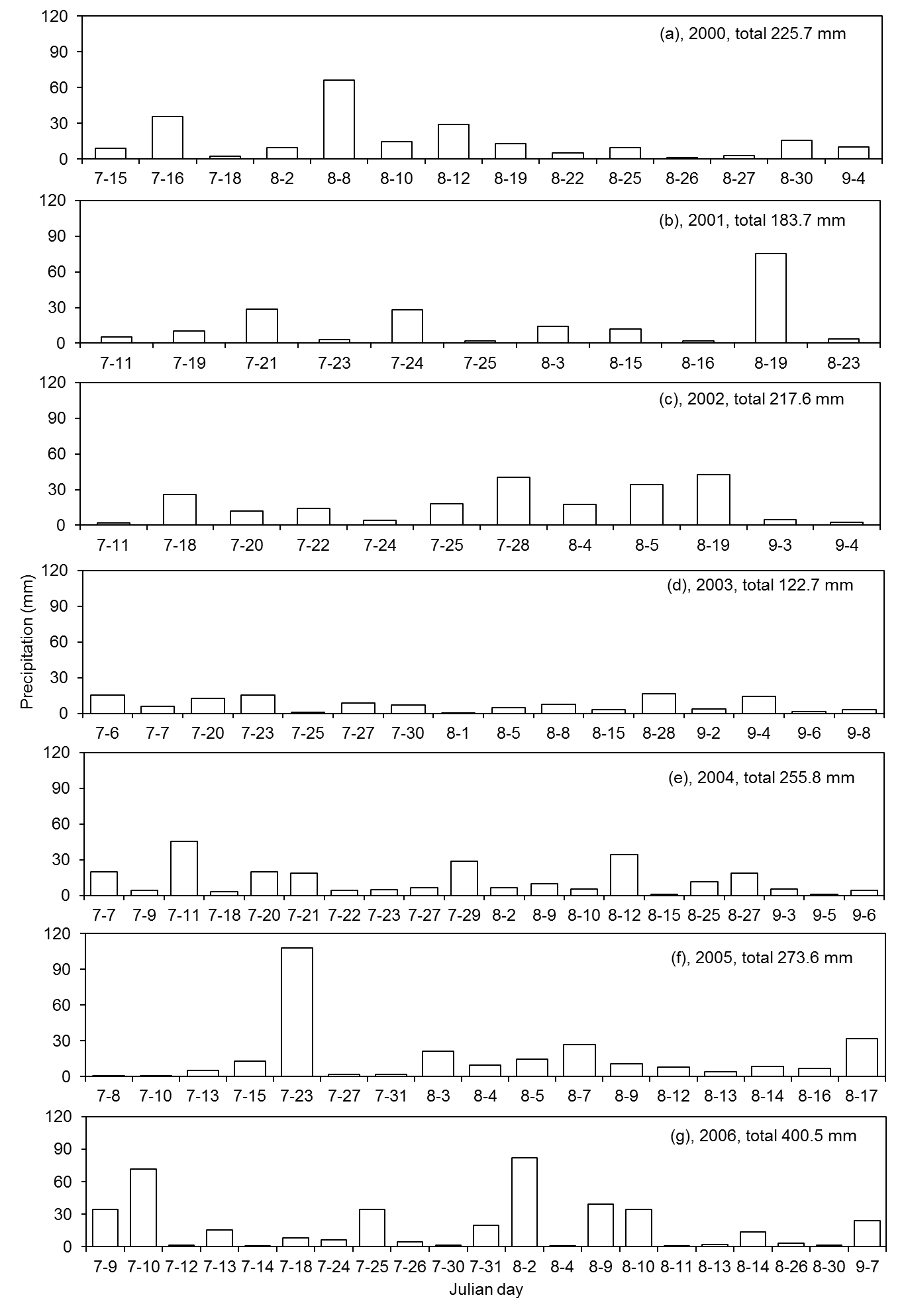


**Fig. S3**. **The daily rainfalls on field experiment site from 2000 to 2006 in Dongbeiwang, Haidian District, suburb of Beijing during the rainfall season lasted from early July to early September**.

**Supplementary Literature Cited**

1. Zhang, W.F. *et al*. New technologies reduce greenhouse gas emissions from nitrogenous fertilizer in China. *Proc. Natl. Acad. Sci.* USA **110,** 8375-8380 (2013).

2. Ye, L. *et al*. Characteristics of nitrate accumulation and its effects on groundwater under typical cropping systems in North China Plain. *J. Soil Water Conserv.* **24,** 165-168,178 (2010) (in Chinese with English abstract).

3.Yuan, H.J. Denitrification in the deep soil from intensive farmlands in the North China Plain. PHD thesis, Center for Agricultural Resources Research, Institute of Genetics and Developmental Biology, CAS (2015) (in Chinese with English abstract).

4. Fu, Y.Q., Lei, Y.P., Zheng, L. & Geng, Q.G. Nitrate leaching characteristics in deep soil layer for different fertilization methods. *Chin. Agric. Sci. Bull.* **22,** 245-248 (2006) (in Chinese with English abstract).

5. Li, X.X., Hu, C.S., Lei, Y.P. & Zhang, Y.M. Calculation for the accumulation of nitrate nitrogen in a Luancheng soil by geoprobe. *Chin. J. Soil Sci.* **37,** 906-908 (2006) (in Chinese with English abstract).

6. Fu, Y.Q., Lei, Y.P., Zheng, L. & Zhang, L. Characteristics of nitrate distribution in deep unsaturated zone in farmland. *Agric. Res. Arid Areas* **14,** 73-76 (2006) (in Chinese with English abstract).

7. Li, S.Q. *et al*. Soil nitrogen pool not to be ignored residual NO_3_^-^-N accumulated in soil profile in semi-arid and semi-humid agro-ecological system. *Agric. Res. Arid Areas* **22,** 1-13 (2004) (in Chinese with English abstract).

8. Guo, S.L. & Che, S.G. Effect of land use change from orchard to farmland on the nitrate accumulation in caliche soils in the Loess Plateau. *Plant Nutrition and Fertilizer Science***15**, 1037-1043(2009) (in Chinese with English abstract).

**Extended reference list for data collection, including 206 references with 7587 observations**

1. Ju, X.T., Liu, X.J. & Zhang, F.S. Accumulation and movement of NO_3_^-^-N in soil profile in winter wheat-summer maize rotation system. *Acta Pedol. Sin.* **40,** 538-546 (2003) (in Chinese with English abstract).

2. Liu, H.B., Li, Z.H., Zhang, Y.G., Zhang, W.L. & Lin, B*.* Characteristics of nitrate distribution and accumulation in soil profiles under main agro-land use types in Beijing. *Sci. Agric. Sin.* **37,** 692-698 (2004) (in Chinese with English abstract).

3. Huang, S.M., Bao, D.J., HuangPu, X.R. & Zhang, H.C. Study on distribution of Nitrate-N in Chao soil and reasonable application of N fertilizer under the crop rotation system of winter wheat and corn. *Soil. Environ Sci* **8,** 271-273 (1999) (in Chinese with English abstract).

4. Zhang, X.M. & Wu, W.L. Study on the nitrate accumulation in the rooting soil profile of high-yield farm ecosystem. *J. China Agric. Univ.* **3,** 106 (1998) (in Chinese with English abstract).

5. Li, S.Q. *et al*. Soil nitrogen pool not to be ignored residual NO_3_^-^-N accumulated in soil profile in semi-arid and semi-humid agro-ecological system. *Agric. Res. Arid Areas* **22**, 1-13 (2004) (in Chinese with English abstract).

6. Kou, C.L., Ju, X.T. & Zhang, F.S. Nitrogen balance and its effects on Nitrate-N concentration of groundwater in three intensive cropping systems of North China. *Chin. J. Appl. Ecol.* **16,** 660-667 (2005) (in Chinese with English abstract).

7. Wu, J.H., Guo, S.L. & Dang, T.H. Mechanisms in the accumulation and movement of mineral N in soil profiles of farming land in a semi-arid region. *Acta Ecol. Sin.* **23,** 2041-2049 (2003) (in Chinese with English abstract).

8. Gu, Q.Z., Yang, X.Y., Sun, B.H, & Ma, L.J. Effect of long-term fertilization on distribution and accumulation of NO_3_^-^-N in loess profile of dry-land. *Agric. Res. Arid Areas* **21,** 48-52 (2003) (in Chinese with English abstract).

9. Dang, T.H., Guo, S.L. & Hao, M.D. The amount and ratio of NO_3_^-^-N accumulation under long-term fertilization in dry highland of Loess Plateau. *Res. Soil Water Conserv.* **10,** 58-61 (2003) (in Chinese with English abstract).

10. Fan, J., Shao, M.A., Hao, M.D. & Wang, Q.J. Nitrate accumulation and distribution in soil profiles in ecosystem of upland on the Loess Plateau. *Plant Nutr. Fert. Sci.* **11,** 8-12 (2005) (in Chinese with English abstract).

11. Li, S.Q., Gao, Y.J., Du, J.J. & Li, S.X. Effect of continuously applying nitrogen fertilizer on the nitrogen in dryland soil. *Agric. Res. Arid Areas* **17,** 28-34 (1999) (in Chinese with English abstract).

12. Yuan, X.M., Li, X.L., Zhang, F.S. & Tong, Y.A. Changes of Nitrate-N accumulated in soil profile after changing grain crop land into vegetables land. *Eco-agriculture Research* **8,** 31-33 (2000) (in Chinese with English abstract).

13. Fan, J., Shao, M.A., Hao, M.D. & Wang, Q.J. Desiccation and nitrate accumulation of apple orchard soil on the Weibei dryland. *Chin. J. Appl. Ecol.* **15,** 1213-1216 (2004) (in Chinese with English abstract).

14. Dang, J.X., Guo, W.L., Guo, J.W., Lv, J.L. & Wang, J.L. Study of the regularity of the salt accumulation of topsoil and NO_3_^-^-N migration in greenhouse soil and years of vegetables cultivation. *Chin. Agric. Sci. Bull.* **20,** 189-191 (2004) (in Chinese with English abstract).

15. Li, Z.H., Liu, H.B., Zhang, S.L. & Zhang, F.S. Soil-plant buffer power for nitrogen fertilizer under wheat-corn rotation. *Sci. Agric. Sin.* **34,** 637-643 (2001) (in Chinese with English abstract).

16. Tong, Y.A., Shi, W., Lv, D.Q. & Emteryd, Ove. Relationship between soil texture and nitrate distribution and accumulation in three types of soil profile in Shaanxi. *Plant Nutr. Fert. Sci.* **11,** 435-441 (2005) (in Chinese with English abstract).

17. Zhang, Y.G., Liu, H.B., Li, Z.H., Lin, B. & Zhang, F.D. Study of nitrate leaching potential from agricultural land in Northern China under long-term fertilization conditions. *Plant Nutr. Fert. Sci.* **11,** 711-716 (2005) (in Chinese with English abstract).

18. Ye, Y.L., Li, L., Sun, J.H. & Zhang, F.S. Effect of root separation on plant nitrogen uptake and soil nitrate nitrogen residual in faba bean/maize intercropping. *J. Soil Water Conserv.* **19,** 13-16, 53 (2005) (in Chinese with English abstract).

19. Wu, Y.C., Zhou, S.L., Wang, Z.M. & Luo, Y.Q. Dynamics and residue of soil nitrate in summer maize field of North China. *Acta Ecol. Sin.* **25,** 1620-1625 (2005) (in Chinese with English abstract).

20. Xu, F.L., Liang, Y.L., Zhang, C.E., Du, S.N. & Chen, Z.J. Effect of fertilization on distribution of nitrate in cucumber and soil in sunlight greenhouse. *Plant Nutr. Fert. Sci.* **10,** 68-72 (2004) (in Chinese with English abstract).

21. Qin, Q.Y. *et al*. The status quo of Nitrate-N accumulation in vegetables protected fields in Guanzhong area of Shaanxi province. *Journal of Yangtze University (Nat Sci Edit)* **2,** 12-15 (2005) (in Chinese with English abstract).

22. Gao, Y.J. *et al*. Effect of fertilization and irrigation on residual Nitrate-N in Soil. *J. Soil Water Conserv.* **19,** 61-64 (2005) (in Chinese with English abstract).

23. Wang, Z.H., Zong, Z.Q., Li, S.X. & Chen, B.M. Nitrate accumulation in vegetables and its residual in vegetable fields. Chin. J. *Envir. Sci.* **23,** 79-83 (2002) (in Chinese with English abstract).

24. Yuan, X.M., Tong., Y.A., Yang., X.Y., Li., X.L. & Zhang., F.S. Effect of organic manure on soil nitrate nitrogen accumulation. *Soil. Environ Sci* **9,** 197-200 (2000) (in Chinese with English abstract).

25. Yang, Z.P., Zhou, H.P., Zhang, Q., Guan, C.L. & Cheng, B. Effects of different fertilizer application countermeasures on the soil nitrate nitrogen accumulation in dryland maize. *Chin. J. Eco-Agric.* **14,** 122-124 (2006) (in Chinese with English abstract).

26. He, W.S., Wang, J.L. & Li, J. Spatial variability of soil mineral nitrogen of farmlands

in different regions of Ningxia. *Agric. Res. Arid Areas* **23,** 47-55 (2005) (in Chinese with English abstract).

27. Huang, S.M., Zhang, H.C., Bao, D.J. & HuangPu, X.R. Effect of applying fertilizer on content and distribution of NO_3_^-^-N in Chao soil and reasonable fertilizer application. *Soil. Environ Sci* **9,** 201-203 (2000) (in Chinese with English abstract).

28. Chen, X.Q., Yao, J., Zhu, W.Q. & Zhang, X.J. The changes of soil nitrate in facility vegetables and its effect on the environment. *Ningxia Journal of Agriculture and Forestry Science and Technology* **5,** 4-5 (2004) (in Chinese with English abstract).

29. Yao, C.X., Chen, Z.L., Lu, L.M., Zhang, J. & Xu, S.Y. Nitric nitrogen in soils of vegetable garden in Shanghai city. *Ecol. ＆Environ.* **14,** 220-223 (2005) (in Chinese with English abstract).

30. Yuan, X.M., Li, X.L., Tong, Y.A. & Zhang, F.S. Nitrate-N accumulation in vegetable soil in Guanzhong area of Shaanxi province. *Journal of Soil Erosion and Soil and Water Conservation* **5,** 102-105 (1999) (in Chinese with English abstract).

31. Liu, F.C., Nie, J.H., Liu, C.S., Fu, L.G. & Xiao, Q.S. Effect of fertilizer application on vertical distribution of NO_3_^-^-N in soil depth profile. *Chin. J. Soil Sci.* **36,** 50-53 (2005) (in Chinese with English abstract).

32. Hu, L.F., Hu, C.S., An, Z.M., Zhang, A.J. & Chen, F. Effects of different tillage patterns on crop yields and nitrate leaching in soil. *J. Soil Water Conserv.* **19,** 186-189 (2005) (in Chinese with English abstract).

33. Yang, S.M. *et al*. Effect of long-term fertilization on soil productivity and nitrate accumulation in Gansu oasis. *Sci. Agric. Sin.* **38,** 2043-2052 (2005) (in Chinese with English abstract).

34. Chen, X., Zhang, Q.Z., Lu, C.Y., Shi, Y. & Zhang, L. Accumulation of soil inorganic nitrogen in mono-harvesting farmlands of northeast China in late autumn. *Chin. J. Appl. Ecol.* **15,** 1887-1890 (2004) (in Chinese with English abstract).

35. Fan, B.Q., Hu, C.F. & Ping, J.L. Effect of irrigation and fertilization on nitrate leaching in loamy fluvo-aquic soil. *Plant Nutr. Fert. Sci.* **4,** 16-21 (1998) (in Chinese with English abstract).

36. Zhang, G.Y. *et al*. NO_3_^-^-N content and distribution of soil under protective vegetable culture. *J. Hebei Agric. Sci.* **8,** 22-25 (2004) (in Chinese with English abstract).

37. Liu, X.J., Ju, X.T., Zhang, F.S., Pan, J.R. & Christie, P. Nitrogen dynamics and budgets in a winter wheat-maize cropping system in the North China Plain. *Field. Crop. Res.* **83,** 111-124 (2003).

38. Kou, C.L., Ju, X.T., Gao, Q., Zhen, L. & Zhang, F.S. Effects of fertilization on soil quality in two different cropping systems. *Acta Ecol. Sin.* **24,** 2548-2556 (2004) (in Chinese with English abstract).

39. Yang, R. & Su, Y.Z. Effects of farmland use type and winter irrigation on nitrate accumulation in sandy farmland soil. *Chin. J. Appl. Ecol.* **20,** 615-623 (2009) (in Chinese with English abstract).

40. Qin, Q.Y., Jia, C.Z., Tong, Y.A., Qu, D. & Tian, Y.B. Effect of nitrogen dose on nitrate-nitrogen accumulation in soil in protected cultivation. *J. Anhui Agric. Sci.* **35,** 152-153 (2007) (in Chinese with English abstract).

41. Yang, Y.H. & Zhang, R.Z. Effects of nitrogen rate on the distribution and accumulation of soil residual nitrate in the rain-fed agricultural region of the middle Loess Plateau. *Chin. J. Soil Sci.* **38,** 672-676 (2007) (in Chinese with English abstract).

42. Nan, W.G., Li, S.Q., Hou, H.Q. & Li, S.X. Dynamic changes of soil NO_3_^-^-N as affected by different N fertilizer application and weed treatments in semi-humid winter wheat field. *Plant Nutr. Fert. Sci.* **14,** 71-78 (2008) (in Chinese with English abstract).

43. Zhou, B., Zhou, J.B., Han, D.F. & Liu, H. Study of nitrate migration in leisure period of greenhouse. *Acta Agric. Bor-Occid. Sin.* **17,** 118-121 (2008) (in Chinese with English abstract).

44. Fan, Y.N., Li, S.Q. & Li, S.X. Utilization rate of fertilizer N and dynamic changes of soil NO_3_^-^-N in summer maize field in semi-humid area of Northwest China. *Chin. J. Appl. Ecol.* **19,** 799-806 (2008) (in Chinese with English abstract).

45. Guo, J.H., Zhao, C.J., Meng, Z.J., Wang, X. & Ma, W. The effect of nitrogen on nitrate leaching and absorption under spring corn in dry arears of North China. *Chin. J. Soil Sci.* **39,** 562-565 (2008) (in Chinese with English abstract).

46. Liu, X.G., Zhang, F.C. & Tian, Y.F. Effects of alternative furrow irrigation on transport of water and nitrogen in maize root zone. *Sci. Agric. Sin.* **41,** 2025-2032 (2008) (in Chinese with English abstract).

47. Yi, W.P. *et al*. Effects of coated controlled release urea combined with conventional urea on winter wheat growth and soil NO_3_^-^-N. *Chin. J. Appl. Ecol.* **22,** 687-693 (2011) (in Chinese with English abstract).

48. Su, T., Wang, Z.H., Zai, S.M. & Li, S.X. Effect of fallow and fertilization on soil mineral nitrogen in summer maize. *Chin. J. Eco-Agric.* **16,** 1078-1082 (2008) (in Chinese with English abstract).

49. Yang, R. & Su, Y.Z. Effects of nitrogen fertilization and irrigation rate on grain yield, nitrate accumulation and nitrogen balance on sandy farmland in the marginal oasis in the middle of Heihe River basin. *Acta Ecol. Sin.* **29,** 1459-1469 (2009) (in Chinese with English abstract).

50. Du, H.X., Wu, P.T., Wang, B.Q., Feng, H. & Ma, J.Y. Influence of applying phosphorus on soil nitrate N and nutrient uptake and yield of summer maize. *J. Northwest Sci-Tech Univ. Agric. For. (Nat. Sci. Ed.)* **37,** 121-126 (2009) (in Chinese with English abstract).

51. Zhang, G.L., Ren, T.Z., Li, Z.H., Liu, H.B. & Zou, G.Y. Effects of nitrogen fertilization on nitrate content of radish and soil nitrate leaching. *Plant Nutr. Fert. Sci.* **15,** 877-883 (2009) (in Chinese with English abstract).

52. Wu, Q. *et al*. Effect of vegetable intercropping on the nitrate accumulation in soil profiles and vegetables. *J. Agro-Environ. Sci.* **28,** 1623-1629 (2009) (in Chinese with English abstract).

53. Zhang, A.P. *et al*. Effects of P fertilizer application on yield of spring wheat and characteristics of N absorption and NO_3_^-^-N accumulation in soil profile. *Agric. Res. Arid Areas* **27,** 30-34 (2009) (in Chinese with English abstract).

54. Zuo, H.J. *et al*. Characteristics of soil nitrate nitrogen dynamic variations and accumulation under intensive planting. *Bull. Soil Water Conserv.* **29,** 16-21 (2009) (in Chinese with English abstract).

55. Lv, F.T., Zhang, X.S., Dong, J. & Ren, Q.P. Characteristics of available nutrients accumulation and eluviation in greenhouse soil. *J. Soil Water Conserv.* **23,** 191-194 (2009) (in Chinese with English abstract).

56. Zhao, Y.Y., Xie, Y.S. & Hao, M.D. Effect of fertilization on fertility and nitrate accumulation of black loessial soil of dry land in Loess Plateau. *Plant Nutr. Fert. Sci.* **15,** 1273-1279 (2009) (in Chinese with English abstract).

57. Huang, J., Wang, B.R., Liu, H.B. & Qin, L. Effects of long-term fertilization on accumulation of NO_3_^-^-N in profile of red soil dry land. *Hunan Agric. Sci.* 60-62, 65 (2010) (in Chinese with English abstract).

58. Yin, X.F. *et al*. Nitrate leaching characteristics of wheat-corn rotation farmland in Guanzhong area of Shanxi. *Chin. J. Appl. Ecol.* **21,** 640-646 (2010) (in Chinese with English abstract).

59. Liu, X.Y., Dong, T., Zhang, K., Zhang, H.Y. & Wang, F.L. Distribution and accumulation of mineral nitrogen in apple orchard soils in dry plateau of eastern Gansu province. *Chin. J. Appl. Ecol.* **21,** 796-800 (2010) (in Chinese with English abstract).

60. Ye, L. *et al*. Characteristics of nitrate accumulation and its effects on groundwater under typical cropping systems in North China Plain. *J. Soil Water Conserv.* **24,** 165-168,178 (2010) (in Chinese with English abstract).

61. Feng, L.L., Zhang, F.C., Lei, Y. & Kou, W.P. Effect of irrigation at different growth period and nitrogen fertilizer on transfer of nitrate nitrogen and yield of the spring maize. *China Rural Water and Hydropower*, 54-57, 61 (2010) (in Chinese with English abstract).

62. Zhang, H., Zhou, J.B., Wang, C.Y., Dong, F. & Li, F.J. Effect of cultivation pattern and nitrogen application rate on soil fertility and nitrate accumulation under maize-wheat rotation system. *Chin. J. Eco-Agric.* **18,** 693−697 (2010) (in Chinese with English abstract).

63. Ning, J.F. *et al*. Effects of nitrogen application rate on the growth of mustard and nitrate residue in soil. *Chin. J. Trop. Crops* **31,** 1329-1334 (2010) (in Chinese with English abstract).

64. Lu, H.N., Tang, Z.J., Feng, S.Y., Zhang, Z.J. & Zhao, L. Spatial variability of nitrate content in different soil layers in the Shunyi district of Beijing. *Acta Sci. Circumst.* **30,** 2563-2570 (2010) (in Chinese with English abstract).

65. Yang, J.G. *et al*. Effects of applying controlled-release fertilizer blended with conventional nitrogen fertilizer on Chinese cabbage yield and quality as well as nitrogen losses. *Chin. J. Appl. Ecol.* **21,** 3147-3153 (2010) (in Chinese with English abstract).

66. Jiang, H.M, *et al*. Effects of different treatments of nitrogen fertilizer on yield, quality of tomato and soil NO_3_^-^-N accumulation in vegetable-greenhouse. *J. Agro-Environ. Sci.* **29,** 2338-2345 (2010) (in Chinese with English abstract).

67. Wang, J.Q., Han, B.W. & Liu, S.P. Effects of nitrogen application rates and soil tillage modes on yield of spring maize and NO_3_^-^-N accumulation in soil profile. *Agric. Res. Arid Areas* **29,** 129-135 (2011) (in Chinese with English abstract).

68. Xie, W.Y., Fan, G.S., Zhou, H.P., Guan, C.L. & Yu. J.W. Effect of long-term fertilization placement on distribution and accumulation of NO_3_^-^-N in cinnamon profile of dry-1and. *Acta Agric. Boreali-Sin.* **26,** 180-185 (2011) (in Chinese with English abstract).

69. Zhang, Y.M. *et al*. Effect of fertilization and irrigation on wheat-maize yield and soil nitrate nitrogen leaching in high agricultural yield region in North China Plain. *Chin. J. Eco-Agric.* **19,** 532−539 (2011) (in Chinese with English abstract).

70. Wang, J.Q., Liu, S.P. & Han, B.W. Effects of nitrogen application on nitrogen use efficiency of spring maize and distribution of soil nitrate nitrogen in northwest Hebei province. *J. Soil Water Conserv.* **25,** 138-143 (2011) (in Chinese with English abstract).

71. Wu, X.L., Zuo, Q., Wu, J.X., Han, J.Q. & Zou, G.Y. Study on measures for reducing nitrogen leaching from facility vegetable field. *J. Soil Water Conserv.* **25,** 59-62, 67 (2011) (in Chinese with English abstract).

72. Bai, R., Li, B.Z., Zhang, L.S. & Gao, D.T. Effect of different factors on NO_3_^-^-N accumulation in apple orchards in Weibei area, Shaanxi province. *J. Fruit Sci.* **24,** 411-414 (2007) (in Chinese with English abstract).

73. Li, Y.T., Li, X.Y., Zhao, B.Q., Wang, L.X. & Li, X.P. Effect of controlled-release compounded fertilizers on maize yields and leaching loss of nitrate in soil profile. *China Soils Fert*. 45-48 (2007) (in Chinese with English abstract).

74. Cai, W.T., Chen, F., Cui, Y.H., Lei, J. & Huai, H.J. Effects of different cropping systems on soil inorganic nitrogen accumulation-A case study in Tongzhou district, Beijing. *Plant Nutr. Fert. Sci.* **15,** 1260-1265 (2009) (in Chinese with English abstract).

75. Wang, L.C., Zhao, L.P., Zhu, P., Gao, H.J. & Peng, C. Effects of different fertilizer application regimes on NO_3_^-^-N and NH_4_^+^-N in black soil during spring maize growing season. *J. Northeast For. Univ.* **37,** 85-87 (2009) (in Chinese with English abstract).

76. Yang, H.F. *et al*. Effects of long-term fertilization modes on spatio-temporal distribution and accumulation of soil nitrate nitrogen in solar greenhouse. *Chin. J. Eco-Agric.* **19,** 246−252 (2011) (in Chinese with English abstract).

77. Zhai, C.X. *et al*. Effects of modified starch coated and slow released urea on growth and nitrogen agronomic efficiency winter wheat. *Acta Agric. Boreali-Sin.* **26,** 175-179 (2011) (in Chinese with English abstract).

78. Wang, X.Y. *et al*. Interactive effects of irrigation and nitrogen fertilizer on nitrogen fertilizer recovery and Nitrate-N movement across soil profile in a winter wheat field. *Acta Ecol. Sin.* **28,** 685-694 (2008) (in Chinese with English abstract).

79. Li, L. *et al*. Effects of nitrogen application and irrigation on soil nitrate accumulation, nitrogen balance and use efficiency in summer maize. *Plant Nutr. Fert. Sci.* **16,** 1358-1365 (2010) (in Chinese with English abstract).

80. Sun, Z.M. *et al*. Analysis on distribution of NO_3_^-^-N in soil profile and environmental effect in corn field of different yields. *J. Soil Water Conserv.* **24,** 162-166,181 (2010) (in Chinese with English abstract).

81. Zhao, S.S. Effect of different fertilization and planting patterns on NO_3_^-^-N accumulation in soil around Baiyangdian Lake. Master thesis, Agricultural University of Hebei (2011) (in Chinese with English abstract).

82. Liu, J.P. The law of nitrate transportation and assimilation study in soil-vegetable system under different conditions of water and fertilizer. Master thesis, Agricultural University of Hebei (2008) (in Chinese with English abstract).

83. Wang, C.Y. Water and nutrient use efficiency of crop with different cultivation methods in rotation system on semi-dryland farming. Master thesis, Northwest A&F University (2007) (in Chinese with English abstract).

84. Dong, F. Effects of different cultivation methods and application of nitrogen on nitrogen mineralization and water use efficiency in soil on dryland and semi-dryland farming. Master thesis, Northwest A&F University (2008) (in Chinese with English abstract).

85. Wu, W.Q., He, J.D., Zhang, H.W. & Zhang, J. The effect of nitrogen on the production of waxy corn and soil environment. *Beijing Agric.* 45-47 (2007) (in Chinese with English abstract).

86. Liu, C.C. NO_3_^-^-N concentration in rhizosphere soil solution and leaching characteristics in typical crops. Master thesis, Agricultural University of Hebei (2011) (in Chinese with English abstract).

87. Guo, Q.Z. *et al*. Effect of different fertilizer and water managements on the accumulation of nutrients and salts in soils under sunlight greenhouses with tomato. *J. Northwest Sci-Tech Univ. Agric. For. (Nat. Sci. Ed.)* **36,** 111-117 (2008) (in Chinese with English abstract).

88. Yuan, L.J. Effects of different measures of controlling water and fertilizer supply on biological validity and movement of deep soil nitrate. Master thesis, Agricultural University of Hebei (2009) (in Chinese with English abstract).

89. Lin, X. Fate of fertilizer nitrogen and gaseous N loss in winter wheat-summer maize rotation system in North China Plain. Master thesis, Agricultural University of Hebei (2007) (in Chinese with English abstract).

90. Zhao, B.Z. & Zhang, J.B. Variation of soil NO_3_^-^-N in content and its distribution in soil profile under intensive agriculture in the North China Plain. *Soils* **39,** 760-765 (2007) (in Chinese with English abstract).

91. Yang, K.Q., Hao, M.D., Zang, Y.F., Sun, Z.Y. & Gao, Y.L. Effect of long-term located fertilization on NO_3_^-^-N accumulation in the profile of loessial soil. *Acta Agric. Bor-Occid. Sin.* **20,** 176-180 (2011) (in Chinese with English abstract).

92. Xie, A.K. Nitrate accumulation and related control strategies in the protected field of Beijing suburb. Master thesis, Chinese Academy of Agricultural Sciences (2010) (in Chinese with English abstract).

93. Ru, J.H., Zhang, G.Y., Sun, S.Y., Geng, N. & Wang, L. Effect of different nitrogen application rate on soil nitrate nitrogen distribution, accumulation and crop yields of Northern China Plan. *Acta Agric. Boreali-Sin.* **27,** 172-177 (2012) (in Chinese with English abstract).

94. Miao, Y.F. *et al*. Characteristics of ammonium N and nitrate N accumulation in dryland soil in relation with wheat yield. *Chin. J. Appl. Ecol.* **25,** 1013-1021 (2014) (in Chinese with English abstract).

95. Sun, J. Study on nitrogen utilization and balance of applied fertilizer in vegetable protected soils under greenhouse in Beijing suburb. Master thesis, Chinese Academy of Agricultural Sciences (2012) (in Chinese with English abstract).

96. Xiao, H., Cheng, W.J., Wang, L.Y., Pan, J. & Gao, X.B. Effects of organic manures on dynamic changes of nitrate contents of soil profile in greenhouse. *Plant Nutr. Fert. Sci.* **18,** 106-114 (2012) (in Chinese with English abstract).

97. Bi, X.Q. *et al*. Effects of nitrogen rates on tomato yield and quality and soil nitrate accumulation under drip irrigation in solar greenhouse. *J. Agro-Environ. Sci.* **32,** 2246-2250 (2013) (in Chinese with English abstract).

98. Dong, Y.W. Studies on soil nitrate-nitrogen accumulation and leaching at eggplant fields. PHD thesis, Chinese Academy of Agricultural Sciences (2011) (in Chinese with English abstract).

99. Shen, L.F. Studies of the effects of protected cultivation years and fertilization on soil salts and NO_3_^-^-N accumulation. Master thesis, Chinese Academy of Agricultural Sciences (2012) (in Chinese with English abstract).

100. Ru, S.H. *et al*. Effect of nitrogen application rate on nitrate nitrogen distribution and accumulation in soils in wheat-maize rotation system. *Acta Agric. Boreali-Sin. (Suppl)* ***2*6,** 85-89 (2011) (in Chinese with English abstract).

101. Cheng, D.J., Liu, Y., Gao, R., Fei, L.J. & Jia, L.H. Effect of fertilizer amount on dynamic change characteristic of soil nitrogen under film hole irrigation. *Journal of Irrigation and Drainage* **31,** 38-42 (2012) (in Chinese with English abstract).

102. Shang, F.Z., Yang, P.L. & Ren, S.M. Effects of nitrogen fertilizer application and irrigation level on soil nitrogen leaching and accumulation in deep soil. *Trans. Chin. Soc. Agric. Mach.* **44,** 112-121 (2013) (in Chinese with English abstract).

103. Gao, N.D. *et al*. Effects of the middle and low yielding fields nitrogen application on nitrogen utilization and soil NO_3_^-^-N accumulation of winter wheat. *Jiangsu Agric. Sci.* **40,** 56-59 (2012) (in Chinese with English abstract).

104. Shen, H.J. *et al*. Effects of nitrogen application rate on yield and nitrogen utilization of winter wheat under straw returning condition in Guanzhong irrigation area. *Acta Agric. Bor-Occid. Sin.* **21,** 72-76 (2012) (in Chinese with English abstract).

105. Zhou, J.B., Chen, Z.J., Liu, X.J., Zhai, B.N. & Powlson, D.S. Nitrate accumulation in soil profiles under seasonally open ‘sunlight greenhouses’ in northwest China and potential for leaching loss during summer fallow. *Soil Use. Manage.* **26,** 332-339 (2010).

106. Chen, X.P. *et al*. Critical grain and stover nitrogen concentrations at harvest for summer maize production in China. *Agron. J.* **102,** 289-295 (2010).

107. Liu, J.L. *et al*. Nitrogen fertilization effects on nitrogen balance and use efficiency for film-mulched maize in a semiarid region. *Acta Agr. Scand. B-s. P* **63,** 612-622 (2013).

108. Chen, X.P., Zhang, F.S., Cui, Z.L., Li, F. & Li, J.L. Optimizing soil nitrogen supply in the root zone to improve maize management. *Soil Sci. Soc. Am. J.* **74,** 1367-1373 (2010).

109. Cui, Z.L. *et al*. Regional evaluation of critical nitrogen concentrations in winter wheat production of the North China Plain. *Agron. J.* **101,** 159-166 (2009).

110. Cui, Z.L., Zhang, F.S., Chen, X.P., Li, F. & Tong, Y.P. Using in-season nitrogen management and wheat cultivars to improve nitrogen use efficiency. *Soil Sci. Soc. Am. J.* **75,** 976-983 (2011).

111. Cui, Z.L., Zhang, F.S., Chen, X.P., Dou, Z.X. & Li, J.L. In-season nitrogen management strategy for winter wheat: Maximizing yields, minimizing environmental impact in an over-fertilization context. *Field. Crop. Res.* **116,** 140–146 (2010).

112. Cui, Z.L. *et al*. On-farm evaluation of the improved soil N_min_-based nitrogen management for summer maize in North China Plain. *Agron. J.* **100,** 517-525 (2008).

113. Cui, Z.L. *et al*. On-farm evaluation of winter wheat yield response to residual soil Nitrate-N in North China Plain. *Agron. J.* **100,** 1527-1534 (2008).

114. Chen, X.P., Zhou, J.C., Wang, X.R., Blackmer, A.M. & Zhang, F.S. Optimal rates of nitrogen fertilization for a winter wheat-corn cropping system in northern China. *Commun. Soil Sci. Plan* **35,** 583-597 (2004).

115. Cui, Z.L. *et al*. Soil Nitrate-N levels required for high yield maize production in the North China Plain. *Nutr. Cycl. Agroecosys* **82,** 187-196 (2008).

116. Lv, L.H. The accumulation of nitrate nitrogen in soil profiles in the semiarid and semi-humid area. Master thesis, Northwest A&F University (2004) (in Chinese with English abstract).

117. Zhang, H. Water and nutrient use efficiency in winter wheat-summer maize rotation system under different cultivation pattern on semi-dryland farming. Master thesis, Northwest A&F University (2010) (in Chinese with English abstract).

118. Jiang, H.M. Effects of nitrogen fertilizer management model on nitrogen residue and utilization in greenhouse vegetable soil. PHD thesis, Chinese Academy of Agricultural Sciences (2012) (in Chinese with English abstract).

119. Wang, L.M. Effects of nitrogen management on dry matter translation, nitrogen uptake and soil nitrate-nitrogen accumulation of high-yield wheat. Master thesis, Henan Agricultural University (2012) (in Chinese with English abstract).

120. Liu, R. Nutrient balance and fertilization for wheat/maize rotation system in western Guanzhong. Master thesis, Northwest A&F University (2011) (in Chinese with English abstract).

121. Wang, X.N. Soil mineral nitrogen and its effects on crop growth and nutrient use in winter wheat and summer maize rotation on dryland. Master thesis, Northwest A&F University (2005) (in Chinese with English abstract).

122. Kou, C.L. Effects of nitrogen fertilization of different intensive cropping systems on environment in North China Plain. PHD thesis, China Agricultural University (2004) (in Chinese with English abstract).

123. Dang, T.H. Accumulative mechanism, crop utilization and environmental response of nitrate in soil profile in dryland on the Loess Plateau. PHD thesis, Northwest A&F University (2005) (in Chinese with English abstract).

124. Yang, Y.H. Effects of continously applying nitrogen on soil nitrate accumulation in rain-fed agriculture region of mid Loess Plateau. Master thesis, Gansu Agricultural University (2006) (in Chinese with English abstract).

125. Zhao, Y. Study on the characteristics of N accumulation and leaching in different farmlands in the Yellow River irrigation region of Ningxia. PHD thesis, Chinese Academy of Agricultural Sciences (2012) (in Chinese with English abstract).

126. Ji, Y.Z. Catch crop control soil nitrogen accumulation and leaching in greenhouse. PHD thesis, Agricultural University of Hebei (2010) (in Chinese with English abstract).

127. Shi, W. Nitrate leaching and nitrogen use under wheat and maize plantation. Master thesis, Northwest A&F University (2005) (in Chinese with English abstract).

128. Fan, Y. The state of organic manure utilization in present and its effects to soil environment. Master thesis, Henan Agricultural University (2006) (in Chinese with English abstract).

129. Wang, H.F., Gao, Z.L., Chen, X.P. & Liu, S.Q. Effects of the combined application of control-released urea and urea on tillers, yield, soil NO_3_^-^-N and nitrogen balance of winter wheat. *Acta Agric. Boreali-Sin.* **27,** 196-201 (2012) (in Chinese with English abstract).

130. Guo, S.L. & Che, S.G. Effect of land use change from orchard to farmland on the nitrate accumulation in caliche soils in the Loess Plateau. *Plant Nutr. Fert. Sci.* **15,** 1037-1043 (2009) (in Chinese with English abstract).

131. Guo, S.L., Hao, M.D. & Dang, T.H. NO_3_^-^-N accumulation and its affecting factors in small watershed in gully region of Loess Plateau. *J. Nat. Resour.* **18,** 37-43 (2003) (in Chinese with English abstract).

132. Zhang, M., Li, L.K. & Hao, M.D. Effect of no-tillage with straw cover on corn yield and soil fertility. *Acta Agric. Bor-Occid. Sin.* **22,** 67-72 (2013) (in Chinese with English abstract).

133. Ran, W., Xie, Y.S. & Hao, M.D. Mineral N accumulation and distribution of apple orchard soil on the Weibei dryland. *Agric. Res. Arid Areas* **26,** 157-160 (2008) (in Chinese with English abstract).

134. Li, X.X., Hu, C.S., Zhang, Y.M., Dong, W.X. & Ouyangzhi, Y. Losses of nitrate-nitrogen from a wheat-corn rotation in north China. *Agric. Res. Arid Areas* **24,** 7-10, 28 (2006) (in Chinese with English abstract).

135. Zhong, Q., Ju, X.T. & Zhang, F.S. Analysis of environmental endurance of winter wheat/summer maize rotation system to nitrogen in North China Plain. *Plant Nutr. Fert. Sci.* **12,** 285-293 (2006) (in Chinese with English abstract).

136. Liu, X.J., Ju, X.T. & Zhang, F.S. Effect of basal application of urea on inorganic nitrogen in soil profile. *J. China Agric. Univ.* **6,** 63-68 (2001) (in Chinese with English abstract).

137. Liu, X.J., Ju, X.T. & Zhang, F.S. Effect of reduced N application on N utilization and balance in winter wheat-summer maize cropping system. *Chin. J. Appl. Ecol.* **15,** 458-462 (2004) (in Chinese with English abstract).

138. Yu, S.F., Yang, L., Zhang, M., Wu, W.L. & Ju, X.T. Effect of controlled release fertilizers on the biological properties of wheat and corn and soil nitrate accumulation. *J. Agro-Environ. Sci.* **29,** 128-133 (2010) (in Chinese with English abstract).

139. Yuan, L.J., Ju, X.T., Zhang, L.J., Wang, J. & Yang, Z.X. NPK accumulation in greenhouse soil and its effect on groundwater. *Chin. J. Eco-Agric.* **18,** 14-19 (2010) (in Chinese with English abstract).

140. Zhang, L.J., Ju, X.T., Ji, Y.Z., Zhang, F.S. & Peng, Z.P. Effects of fallow and plant growth in summer on the movement of residual nitrate in aquic soil on North China Plain. *Plant Nutr. Fert. Sci.* **16,** 312-320 (2010) (in Chinese with English abstract).

141. Wang, X.M., Xie, Y.X., Zhang, Y.N., Zhu, Y.J. & Yan, X.Y. Effect of new type fertilizers application on accumulation of soil nitrate nitrogen in the maize season. *J. Soil Water Conserv.* **23,** 232-236 (2009) (in Chinese with English abstract).

142. Li, J.L. *et al*. Nitrate leaching loss from soil and nutrient utilization by tomato in protected field. *Chin. J. Appl. Environ. Biol.* **7,** 126-129 (2001) (in Chinese with English abstract).

143. Liu, K., Chen, X.P. & Zhang, F.S. Winter wheat root distribution and soil water and nutrient availability. *Acta Pedol. Sin.* **40,** 697-703 (2003) (in Chinese with English abstract).

144. Zhou, S.L., Zhang, F.S. & Wang, X.R. Study on differences in nitrogen nutrition between winter wheat varieties in response, uptake to nitrogen and associated depletion of soil nitrate. *Sci. Agric. Sin.* **35,** 667-672 (2002) (in Chinese with English abstract).

145. Cui, Z.L. *et al*. Appropriate soil nitrate N content for a winter wheat/summer maize rotation system in North China Plain. *Chin. J. Appl. Ecol.* **18,** 2227-2232 (2007) (in Chinese with English abstract).

146. Zhu, J.H., Li, J.L., Li, X.L. & Zhang, F.S. Effects of compound fertilizers utilized on soil environmental quality in protected vegetable field. *Agro-Environ. Prot.* **21,** 5-8 (2002) (in Chinese with English abstract).

147. Zhou, S.L., Zhang, F.S. & Wang, X.R. Studies on the spatio-temporal variations of soil NO_3_^-^-N and apparent budget of soil nitrogenⅠ.winter wheat. *Acta Ecol. Sin.* **21,** 1782-1789 (2001) (in Chinese with English abstract).

148. Wang, X.L., Li, L., Jiang, R.F. & Zhang, F.S. Effects of maize/swamp cabbage intercropping on reduction of the nitrate content in soil profile and vegetables. *Acta Sci. Circumst.* **23,** 463-467 (2003) (in Chinese with English abstract).

149. Wang, C.Y., Zhou, J.B., Zheng, X.F. & Li, S.X. Effects of different cultivation methods on soil residual nitrate under winter wheat-summer maize cropping system. *Plant Nutr. Fert. Sci.* **13,** 991-997 (2007) (in Chinese with English abstract).

150. Liu, X.J., Chen, Z.J., Zhang, Y.L. & Zhou, J.B. Nutrient accumulation in the sunlight greenhouse soils with the different cultivating years. *Chin. J. Soil Sci.* **40,** 285-289 (2009) (in Chinese with English abstract).

151. Zhou, J.B. *et al*. Mineral nitrogen content and accumulation in soil profiles under sunlight greenhouse cultivation. *Plant Nutr. Fert. Sci.* **12,** 675-680 (2006) (in Chinese with English abstract).

152. Zhou, J.B., Zhai, B.N., Chen, Z.J., Ma, A.S. & Shang, H.B. Nutrient accumulations in soil profiles under canopy vegetable cultivation and their potential environmental impacts. *J. Agro-Environ. Sci.* **23,** 332-335 (2004) (in Chinese with English abstract).

153. Gao, J.J., Lei, J.F., Chen, Z.J. & Zhou, J.B. Effects of fertilization on the yield of tomato and soil nutrient contents in newly-built sunlight greenhouse. *Agric. Res. Arid Areas* **30,** 19-24 (2012) (in Chinese with English abstract).

154. Bai, X.L., Gao, J.J., Chen, Z.J., Lei, J.F. & Zhou, J.B. Nutrient accumulation and balances in soils of the new-established greenhouses. *China Soils Fert*. 1-5 (2014) (in Chinese with English abstract).

155. Zhang, Z.L. *et al*. Nitrogen recommendation for dryland winter wheat by monitoring nitrate in 1 m soil and based on nitrogen balance. *Plant Nutr. Fert. Sci.* **18,** 1387-1396 (2012) (in Chinese with English abstract).

156. Su, T., Wang, Z.H. & Li, S.X. Relations between water and temperature factors and dynamics of soil mineral nitrogen during summer maize growing season. *Chin. J. Soil Sci.* **42,** 896-901 (2011) (in Chinese with English abstract).

157. Chen, H.L., Liu, Y., Tian, X.H., Zhao, A.Q. & Wang, Z.H. Effects of different cultivation modes on soil water, yield and NO_3_^-^-N accumulation in Weibei Loess Plateau. *Agric. Res. Arid Areas* **29,** 19-25 (2011) (in Chinese with English abstract).

158. Wang, X.N., Wang, Z.H. & Li, S.X. Influence of planting maize and fallowing on soil moisture and mineral nitrogen. *Sci. Agric. Sin.* **39,** 1179-1185 (2006) (in Chinese with English abstract).

159. Cai, W.T., Chen, F., Zhang, H.L., Lei, J. & Wen, X.Y. Effects of different nitrogen fertilization levels on yield of cucumber, the soil Nitrate-N accumulation and the soil solution Nitrate-N concentrations. *Acta Agric. Boreali-Sin.* **24,** 189-193 (2009) (in Chinese with English abstract).

160. Zhao, J.Y. & Yu, Z.W. Effects of nitrogen rate on nitrogen fertilizer use of winter wheat and content of soil Nitrate-N under different fertility condition. *Acta Ecol. Sin.* **26,** 815-822 (2006) (in Chinese with English abstract).

161. Zhang, X.J., Zhao, Y., Chen, X.Q., Wu, L.S. & Hu, C.X. Effects of application of nitrogen on vegetable yield, nitrogen balance and soil nitrogen accumulation under two years’ drip fertigation. *Sci. Agric. Sin.* **40,** 2535-2545 (2007) (in Chinese with English abstract).

162. Li, J.M., Li, S.J., Zeng, C.L. & Zhou, D.X. Studies on the soil nitrate content and nitrogen balance in winter wheat plants under limited irrigation condition. *Acta Agric. Boreali-Sin.* **18,** 51-55 (2003) (in Chinese with English abstract).

163. Lv, D.Q., Yang, J.R. & Ma, L.Y. Study on effect of irrigation on soil nitrate leaching and uptake. *Plant Nutr. Fert. Sci.* **5,** 307-315 (1999) (in Chinese with English abstract).

164. Zhao, P. & Chen, F. Effects of straw mulching and nitrogen fertilizer application on nitrogen use in summer maize and soil nitrate content. *J. Henan Agric. Univ.* **43,** 14-18 (2009) (in Chinese with English abstract).

165. Liu, H.B., Li, Z.H., Zhang, W.L. & Li, B. Study on N use efficiency of Chinese cabbage and nitrate leaching under open field cultivation. *Plant Nutr. Fert. Sci.* **10,** 286-291 (2004) (in Chinese with English abstract).

166. Shi, Y. & Yu, Z.W. Effects of nitrogen fertilizer rate and ratio of base and topdressing on yield of wheat, content of soil nitrate and nitrogen balance. *Acta Ecol. Sin.* **26,** 3661-3669 (2006) (in Chinese with English abstract).

167. Xie, H.M., Zhu, B. & Zhu, Z.L. Temporal-spatial variations of soil ammonia and nitrate under application of inorganic and organic fertilizers in purple soil-Summer corn season. *Chin. J. Eco-Agric.* **14,** 103-106 (2006) (in Chinese with English abstract).

168. Zhou, J.B., Zhai, B.N., Chen, Z.J., Xu, A.M. & Feng, W.H. Fertilizers application and nutrient accumulations in tomato-grown soils under greenhouse condition in the suburban of Xian city. *Chin. J. Soil Sci.* **37,** 287-290 (2006) (in Chinese with English abstract).

169. Guo, S.L., Wu, J.S., Hao, M.D. & Dang, T.H. Effect of long-term fertilization on NO_3_^-^-N accumulation and moisture distribution in soil profiles. *Chin. J. Appl. Ecol.* **14,** 75-78 (2003) (in Chinese with English abstract).

170. Fu, Y.Q., Lei, Y.P., Zheng, L. & Zhang, L. Characteristics of nitrate distribution in deep unsaturated zone in farmland. *Agric. Res. Arid Areas* **14,** 73-76 (2006) (in Chinese with English abstract).

171. Fu, Y.Q., Lei, Y.P., Zheng, L. & Geng, Q.G. Nitrate leaching characteristics in deep soil layer for different fertilization methods. *Chin. Agric. Sci. Bull.* **22,** 245-248 (2006) (in Chinese with English abstract).

172. Zhao, R.F. *et al*. Fertilization and nitrogen balance in a wheat-maize rotation system in North China. *Agron. J.* **98,** 938-945 (2006).

173. Zhang, Y.L. & Ju, X.T. Ming the accumulated nitrate from deep soil layers by rotation with different crops. *Sci. Agric. Sin.* **45,** 3297-3309 (2012) (in Chinese with English abstract).

174. Guo, S.L., Dang, T.H. & Hao, M.D. Mineral N distribution of soil profile in gully area of the Loess Plateau. *Agric. Res. Arid Areas* **18,** 22-27, 37 (2000) (in Chinese with English abstract).

175. Li, X.X., Hu, C.S., Lei, Y.P. & Zhang, Y.M. Calculation for the accumulation of nitrate nitrogen in a Luancheng soil by geoprobe. *Chin. J. Soil Sci.* **37,** 906-908 (2006) (in Chinese with English abstract).

176. Yang, Z.X., Zhou, H.P., Guan, C.L. & Xie, W.Y. Effect of long-term straw returning on distribution and accumulation of nitrate nitrogen in dryland soil. *Acta Agric. Boreali-Sin.* **28,** 179-182 (2013) (in Chinese with English abstract).

177. Zhang, H.X., Zhou, H.P., Yang, Z.X., Xie, W.Y. & Guan, C.L. Effect of long-term fertilization to distribution and accumulation of NO_3_^-^-N in dryland soil. *J. Shanxi Agric. Sci.* **42,** 465-469 (2014) (in Chinese with English abstract).

178. Yuan, H.J. Denitrification in the deep soil from intensive farmlands in the North China Plain. PHD thesis, Center for Agricultural Resources Research, Institute of Genetics and Developmental Biology, CAS (2015) (in Chinese with English abstract).

179. Li, X.X., Hu, C.S. & Chen, Y.S. Effects of different fertilizers on crop yields

and nitrate accumulation. *Agric. Res. Arid Areas* **21,** 38-42 (2003) (in Chinese with English abstract).

180. Yuan, X.M., Yang, X.Y., Tong, Y.A., Li, X.L. & Zhang, F.S. A study on optimal irrigation index for spring wheat and maize in major irrigation area of Inner Mongolia. *Agric. Res. Arid Areas* **19,** 8-13, 39 (2001) (in Chinese with English abstract).

181. Yang, X.Y., Zhang, S.L., Yuan, X.M. & Tong, Y.A. A long-term experiment on effect of organic manure and chemical fertilizer on distribution, accumulation and movement of NO_3_^-^-N in soil. *Plant Nutr. Fert. Sci.* **7,** 134-138 (2001) (in Chinese with English abstract).

182. Tong, Y.A., Lv, D.Q. & Zhang, H. Nitrogen balance and nitrate leaching in soils of irrigation district. *Shaanxi J. Agric. Sci.* 12-13 (1994) (in Chinese with English abstract).

183. Fan, J., Hao, M.D. & Dang, T.H. Distribution and accumulation of NO_3_^-^-N in soil profile of long-term located fertilizer experiment. *Soil. Environ Sci* **9,** 23-26 (2000) (in Chinese with English abstract).

184. Guo, S.L., Dang, T.H. & Hao, M.D. Mineral N distribution in soil profiles of slope land in gully region of Loess Plateau. *J. Soil Water Conserv.* **17,** 31-33 (2003) (in Chinese with English abstract).

185. Li, X.X., Hu, C.S. & Chen, S.Y. Effects of controlled irrigation on nitrate accumulation in North China. *J. Hebei Agric. Sci.* **9,** 6-10 (2005) (in Chinese with English abstract).

186. Yuan, X.M., Tong, Y.A., Yang, X.Y., Li, X.L. & Zhang, F.S. Effect of phosphate application on soil nitrate nitrogen accumulation. *Plant Nutr. Fert. Sci.* **6,** 397-403 (2000) (in Chinese with English abstract).

187. Lv, D.Q., Tong, Y.A. & Sun, B.H. Study on effect of nitrogen fertilizer use on environment pollution. *Plant Nutr. Fert. Sci.* **4,** 8-15 (1998) (in Chinese with English abstract).

188. Dang, T.H., Qi, L.H., Guo, S.L. & Hao, M.D. Relationship between soil nitrate, nitrogen balance and utilization in rainfed land. *Plant Nutr. Fert. Sci.* **15,** 573-577 (2009) (in Chinese with English abstract).

189. Xue, X.H. & Hao, M.D. Mineral nitrogen in soil profiles under long-term wheat cultivation and alfalfa continuous cropping in the dry farming area. *Plant Nutr. Fert. Sci.* **16,** 620-625 (2010) (in Chinese with English abstract).

190. Xu, M.J. N characteristics of crop-soil-fertilizer continuums of different rotation systems. Master thesis, Agricultural University of Hebei (2014) (in Chinese with English abstract).

191. Li, J. Assessment of environmental impacts of nitrogen under different yields and input levels of winter wheat/summer maize rotation on the North China Plain. Master thesis, China Agricultural University (2013) (in Chinese with English abstract).

192. Chou, S.J. Interaction of carbon and nitrogen in soil with high accumulated nitrate on the North China Plain. PHD thesis, China Agricultural University (2010) (in Chinese with English abstract).

193. Huang, T. The effects of long-term C and N inputs on soil organic C and N pools and environments. PHD thesis, China Agricultural University (2014) (in Chinese with English abstract).

194. Ju, X.T. Transformation and fate of soil-fertilizer nitrogen in winter wheat/summer maize rotation system. PHD thesis, China Agricultural University (2000) (in Chinese with English abstract).

195. Gao, Q. Crop availability and fate of residual nitrate in different position of soil profile. PHD thesis, China Agricultural University (2003) (in Chinese with English abstract).

196. Zhao, L.L. Movement of accumulative nitrate affected by strong rainfall and irrigation in summer. Master thesis, China Agricultural University (2007) (in Chinese with English abstract).

197. Wang, D.P. Optimized management of resources and sustainability evaluation for crop production system in the high yield regions of North China. PHD thesis, China Agricultural University (2011) (in Chinese with English abstract).

198. Zhong, Q. Studies of nitrogen environmental endurance of winter wheat/summer maize rotation system in North China Plain. Master thesis, China Agricultural University (2004) (in Chinese with English abstract).

199. Zhao, X.N. Studies on reducing nitrate leaching in intensive agricultural field on the North China Plain. Master thesis, China Agricultural University (2011) (in Chinese with English abstract).

200. Zhang, L,J. The behavior of residue nitrate and its utilization by plants in Agro-ecosystem. PHD thesis, China Agricultural University (2004) (in Chinese with English abstract).

201. Guo, Z.D. Optimizing management of nitrogen to control non-point source pollution in intensive agricultural systems in North China. Master thesis, China Agricultural University (2009) (in Chinese with English abstract).

202. Dong, X.X. N characteristics of soil-crop continuums of different rotation systems. Master thesis, Agricultural University of Hebei (2012) (in Chinese with English abstract).

203. Qiao, J.J. *et al*. Effect of different fertilization modes on quality of ‘cabernet sauvignon’ and soil nitrogen. *Northern Horticulture*, 163-168 (2015) (in Chinese with English abstract).

204. Ji, Y.Z. *et al*. Effects of different fertilization pattern on the yield of the rotation system of wheat and maize and soil nitrate accumulation in North China Plain. *Ecol. ＆ Environ. Sci.* **23,** 1725-1731 (2014) (in Chinese with English abstract).

205. Li, Q.Q. *et al*. Effects of conventional and optimized nitrogen fertilization on spring maize yield, ammonia volatilization and nitrogen balance in soil-maize system. *Plant Nutr. Fert. Sci.* **21,** 571-579 (2015) (in Chinese with English abstract).

206. Hao, X.R. *et al*. Effect of root layer regulations on soil nitrate of the rotation

system of sweet corn-cucumber in greenhouse vegetable production system. *Sci. Agric. Sin.* **48,** 2390-2400 (2015) (in Chinese with English abstract).
